# Parasitoid‐mediated indirect interactions between unsuitable and suitable hosts generate apparent predation in microcosm and modeling studies

**DOI:** 10.1002/ece3.6896

**Published:** 2021-02-23

**Authors:** Lucie S. Monticelli, Nicolas Desneux, George E. Heimpel

**Affiliations:** ^1^ Université Côte d’Azur, INRAE, CNRS UMR ISA Nice France; ^2^ Agroécologie INRAE Univ. Bourgogne Franche‐Comté Dijon France; ^3^ Department of Entomology University of Minnesota Saint Paul MN USA

**Keywords:** *Aphelinuscertus*, *Aphisglycines*, *Aphisnerii*, apparent parasitism, apparent predation, biological control, egg sink, evolutionary trap

## Abstract

Parasitoids used as biological control agents often parasitize more than a single host species and these hosts tend to vary in suitability for offspring development. The population dynamics of parasitoids and hosts may be altered by these interactions, with outcomes dependent on the levels of suitability and acceptance of both host species. Parasitism of individuals of an unsuitable host species may indirectly increase populations of a suitable host species if eggs laid into unsuitable hosts do not develop into adult parasitoids. In this case, the unsuitable host is acting as an egg sink for parasitoids and this can reduce parasitism of suitable hosts under conditions of egg limitation. We studied parasitoid‐mediated indirect interactions between two aphid hosts, *Aphis glycines* (the soybean aphid) and *A*.* nerii* (the milkweed, or oleander aphid), sharing the parasitoid *Aphelinus certus*. While both of these aphid species are accepted by *A*.* certus*, soybean aphid is a much more suitable host than milkweed aphid is. We observed a drastic reduction of parasitoid offspring production (45%) on the suitable host in the presence of the unsuitable host in microcosm assays. *Aphelinus certus* females laid eggs into the unsuitable hosts (*Aphis nerii*) in the presence of the suitable host leading to egg and/or time limitation and reduced fitness. The impact of these interactions on the equilibrium population sizes of the three interacting species was analyzed using a consumer–resource modeling approach. Both the results from the laboratory experiment and the modeling approaches identified *apparent predation* between soybean aphid and milkweed aphid, in which milkweed aphid acts as a sink for parasitoid eggs leading to an increase in the soybean aphid population. The presence of soybean aphids had the opposite effect on milkweed aphid populations as it supported increases in parasitoid abundance and thus reduced the fitness and abundance of this aphid species.

## Graphical abstract

We investigated indirect interactions that can occur when a single parasitoid species attacks two aphid species that differ in their suitability for parasitism. Laboratory studies showed that the presence of unsuitable hosts indirectly increased populations of suitable hosts through an egg sink experienced by the shared parasitoid, and a parameterized population model confirmed and extended this result to longer time scales. These results show how such indirect effects can affect community structure and affect biological control interactions.

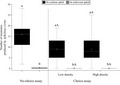

## 1 INTRODUCTION

Parasitoids are insects whose females deposit eggs in, on or near host individuals upon which their larvae develop (Godfray, 1994). Hosts attacked by parasitoids of a given species may range from highly suitable if a majority of parasitoid individuals can complete their development to unsuitable when hosts induce death of immature parasitoids due to physiological factors such as encapsulation (Strand & Pech, 1995), sequestration of toxic compounds (Ode, 2006), and/or the presence of defensive endosymbionts (Oliver et al., 2003). In the case of partially or wholly unsuitable hosts, parasitized individuals may or may not survive parasitism (Kaser et al., 2018). In either case, female parasitoids of a given species often attack multiple host species that vary greatly in suitability (Heimpel et al., 2003; Hopper et al., 2013; Straub et al., 2011) and the diversity of hosts spatially overlapping with parasitoid populations may affect indirect interactions between parasitoids and the hosts of varying suitabilities that they parasitize (Abram et al., 2014, 2016; Heimpel et al., 2003; Hoogendoorn & Heimpel, 2002; Kaser & Heimpel, 2015; Kaser et al., 2018; Kaser & Ode, 2016).

Indirect interactions between two host species sharing a single parasitoid species can be described as a combination of positive, negative or neutral interactions that are experienced by populations of the two host species. Thus, apparent competition is defined as an interaction in which the parasitoid mediates a reciprocal negative interaction between populations of both host species (−/−), apparent predation (or parasitism) is an interaction in which a parasitoid mediates a positive effect for the population of one host species and a negative one for the other (+/−) and apparent mutualism is an interaction in which a parasitoid mediates a positive outcome for populations of both species (+/+) (Abrams, 1987; Chailleux et al., 2014; Holt, 1977; Van Veen et al., 2006). The presence of an unsuitable host species can act as an egg sink for parasitoids if such host individuals are parasitized, leading to an increase in densities of suitable hosts (compared to a situation without the unsuitable host) if parasitoids become egg limited and a decrease in unsuitable hosts (compared to a situation without the suitable host) due to parasitoid enrichment and thus, an apparent predation (indirect ± interaction) scenario (Heimpel et al., 2003). The occurrence of these interactions represents a key mechanism in determining the establishment and strength of food web interactions in ecological systems, including agricultural ones and can have important consequences for biological control outcomes if one of the hosts is a target or nontarget species of a biological control intervention (Emery & Mills, 2020; Heimpel & Mills, 2017; Kaser & Heimpel, 2015; Kaser & Ode, 2016).

Numerous parasitoid species readily attack two or more species that vary in their degree of suitability (reviewed by Heimpel et al., 2003), and in these cases, unsuitable host individuals can act as an egg and/or time sink for the parasitoids leading to reduce parasitism on suitable hosts and an overall decrease in parasitoid populations. These dynamics have been shown or hypothesized for a number of host‐parasitoid systems, including lady beetles and their parasitoids (Hoogendoorn & Heimpel, 2002) and stinkbugs or moths and their egg parasitoids (Abram et al., 2014, 2016; Kaser et al., 2018). Aphids and their parasitoids are also likely candidates for egg sink dynamics based on observations from a number of laboratory studies that aphid species of varying suitability are often parasitized by aphid parasitoid species (e.g., Antolin et al., 2006; Carver, 1984; Desneux et al., 2009a,c; Henry et al., 2010; Hopper et al., 2017a,b; Hopper et al., 2013; Mackauer et al., 1996; Ode et al., 2005).

We investigated direct and indirect interactions between two species of aphid hosts that were known to vary greatly in their suitability for a single parasitoid species under laboratory conditions (Kaser, 2016). We used these studies to determine the magnitude of an egg sink within the unsuitable host and the extent to which this egg sink can benefit the suitable host. We also parameterized an existing mathematical model incorporating egg sink effects (Abram et al., 2016; Heimpel et al., 2003) to investigate the effect of these interactions on population size equilibria for this 2‐host/1‐parasitoid system.

## 2 MATERIALS AND METHODS

### 2.1 The study system

The two hosts investigated as part of this study were *Aphis glycines* as the suitable host species and *Aphis nerii* as the unsuitable host species. *Aphis glycines* is specialized on soybean during summer months and is a key pest on this crop (Ragsdale et al., 2011) while milkweed aphid is specialized on members of the plant family Apocynaceae (Martel & Malcolm, 2004). In North America, this aphid has notably been found on milkweed plants growing inside and outside soybean field (Martin & Burnside, 1980). Soybean aphid is not known to sequester toxic compounds, while milkweed aphid sequesters toxic secondary metabolites (cardenolides) from its host plants which are toxic against natural enemies (Mooney et al., 2008) including parasitoids (Desneux et al., 2009a). However, milkweed aphid has been reported to be attacked by various parasitoid species, including *Aphelinus abdominalis*, *Aphidius ervi*, *Aphidius colemani*, *Binodoxys communis*, *Lysiphlebus testaceipes*, and *Diaeretiella rapae* (Benelli et al., 2014; Colvin & Yeargan, 2013; Cortez‐Madrigal et al., 2016; Desneux et al., 2009a; Hartbauer, 2010; Helms et al., 2004; Monticelli et al., 2019a; Vaz et al., 2004; Wyckhuys & Heimpel, 2007). We used *A*. *certus* in our study because (a) it is known as a parasitoid of soybean aphid, which has been shown to be suitable (Frewin et al., 2010; Heimpel et al., 2010; Hopper & Diers, 2014; Hopper, et al., 2017a; Kaser & Heimpel, 2018) and (b) it is known to attack milkweed aphid despite the low level of suitability of this aphid species (Kaser, 2016). In addition, the potential egg sink caused by the presence of an unsuitable host may be favored by low daily parasitoid fecundity (Heimpel et al., 2003; Kaser et al., 2018), as is the case in *A*.* certus* (Miksanek & Heimpel, 2020a).

*Aphelinus certus* has shown variable efficacy as a biological control agent of the soybean aphid (Hallett et al., 2014; Kaser & Heimpel, 2018; Leblanc & Brodeur, 2018; Miksanek & Heimpel, 2020b). Common milkweed, *Asclepias syriaca*, grows adjacent to (and sometimes within) soybean fields in the United States and it is often infested with *Aphis nerii* (Mohl et al., 2016) although the recent use of glyphosate‐tolerant soybeans has led to declines of this plant in agricultural areas (Stenoien et al., 2018). Understanding the interactions between *A*. *nerii* and *A*.* certus* may shed light on potential indirect effects on populations of the soybean aphid in field settings.

### 2.2 Aphid and parasitoid colonies

*Aphis glycines* and milkweed aphid were reared at the University of Minnesota on soybean (*Glycines max*) and on swamp milkweed (*Asclepias incarnata*) respectively, in growth chambers at 25°C, 16:8h (light:dark) photoperiod, and approximately 65% relative humidity. The *A*. *certus* colony was maintained on its main host, soybean aphid. Before experiments, parasitized aphids were isolated during the mummy stage in 6 cm diameter plastic Petri dishes and newly emerged females were mated and fed with honey solution (50% water + 50% honey) for 24 hr.

To evaluate the indirect interaction between an unsuitable host and a suitable host sharing a parasitoid, it is necessary to compare the direction and strength of the interactions in the absence (no‐choice test) and in the presence (choice test) of the unsuitable host on the parasitoid behavior and successful development. Hence, experiment 1 assessed the indirect interactions between milkweed aphid and soybean aphid, testing the parasitoid's ability to oviposit (experiment 1.1) and the ability of the parasitoid to develop successfully on both species (experiment 1.2) in no‐choice and choice assays, that is, with or without the other species. To evaluate the impact of nonreproductive mortality induced by *A*. *certus* on *Aphis nerii*, experiment 2 assessed the direct impact of parasitoid activity on milkweed aphid fitness comparing the survival and the fecundity of milkweed aphid when it was stung or not by *Aphelinus certus*.

### 2.3 Experiment 1: Parasitoid‐mediated indirect interactions between milkweed aphid and soybean aphid

#### 2.3.1 Experiment 1.1: Parasitoid oviposition

In the no‐choice assay, one leaf of soybean or milkweed (leaves of the two plants were chosen to be of similar size) was cut at the stem and separately placed into a tube (diameter: 2.5 cm, h: 7cm) with 1 cm of watered sand and closed by a perforated cap. Each leaf was then infested with 20 3rd instar *A*. *glycines* (20Ag) or *A*. *nerii* (20An) on soybean and milkweed, respectively (*n* = 25–27 replicates).

In the choice assay, one leaf of soybean and milkweed was cut and placed into the same tube in the same conditions as in the no‐choice assay. The appropriate leaves were then infested with 10 aphids of *A*. *glycines* and *A*. *nerii* (10Ag + 10An) (*n* = 25 replicates) or 20 aphids of each species (20Ag + 20An) onto their respective plant (*n* = 25 replicates). The two densities enabled us to evaluate the impact of aphid abundance on the potential egg sink caused by the presence of the unsuitable host. One mated female parasitoid (between 24 and 48 hr old) was then introduced into the tube of both the no‐choice and choice assays for 24 hr.

After parasitoid removal in both no‐choice and choice assays, aphids were frozen and later dissected at 40× magnification to record the number of eggs laid by the parasitoid into individuals of each aphid species. To determine whether these female parasitoids experienced egg limitation during the assays, 12 randomly chosen females from each of the four different treatments were frozen after they were removed from the arena and later dissected at 40× magnification to count the number of remaining eggs. To make sure there was no systematic bias in size of adults among the treatment, the right hind tibia length was also measured.

#### 2.3.2 Experiment 1.2: Parasitoid developmental success and offspring fitness

In the no‐choice assay, one plant of soybean (10 days old) or milkweed (21 days old) of approximately the same size was potted individually (depth: 13.5 cm, top diameter: 14 cm) and covered in a cylindrical plastic cover (diameter: 11 cm, h: 21 cm) with several 3 cm wide mesh‐covered holes cut in the sides and on the top. Potting soil was covered with white plaster to prevent the development of fungus gnats. The plants were then infested with 50 3rd instar *A*.* glycines* (50 Ag) or *A*.* nerii* (50 An) on soybean and milkweed, respectively (*n* = 18 and 33 plants, respectively).

In the choice assay, single soybean and milkweed plants were potted together under the same conditions as in the no‐choice assay. The appropriate plants were then infested with 25 aphids of each species (25Ag + 25 An) (*n* = 27 replicates) or 50 aphids of each species (50Ag + 50 An) onto their respective plant (*n* = 26 replicates). As in Experiment 1.1, the two different densities enabled us to determine the impact of aphid abundance on the potential egg sink caused by the presence of the unsuitable host. One mated female parasitoid (between 24 and 48 hr old) was then introduced into the covered pots for 24 hr.

In both the no‐choice and choice assays, plants were inspected 10 days later and all mummies were removed and isolated into 0.6 ml microcentrifuge tubes. The total number of mummies and emerged parasitoids were counted and the adult offspring sex ratios were recorded. To determine the effect of the treatments on egg limitation in *A*. *certus*, 46 (n for each of the 4 treatments was 11 or 12) were frozen after the 24 hr of the experiment and later dissected at 40× magnification to count the number of remaining eggs and measure the right tibia length.

## 3 Experiment 2: Impact of parasitism on milkweed aphid

In this experiment, we evaluated mortality of the unsuitable host *A*.* nerii* induced by parasitism by *Aphelinus certus* using a combination of direct observation and rearing. To prepare for observations, a milkweed leaf was placed upside down within a clear plastic cube (1 cm^3^) under a magnifying lens (8×). One second or third instar milkweed aphid was introduced onto the leaf within this cube using a fine brush. Five minutes later, one mated female parasitoid (between 24 and 48 hr old) was also introduced and observed until oviposition occurred (*n* = 37). The same procedure was followed without introducing a parasitoid as a control to quantify background aphid mortality (*n* = 33). Aphids parasitized or not were then placed separately onto milkweed leaves cut at the stem which were then introduced into a tube (diameter: 2.5 cm, h: 7cm) with 1 cm of watered sand at the base (as described above) and closed with a perforated plastic cap. The aphids were observed daily to record their longevity, fecundity, and the period of time before the first nymphs were laid. In order to record daily fecundity, newly produced nymphs were removed every day.

### 3.1 Impact of interactions on shared hosts—parasitoid population dynamics

We used a discrete‐time Nicholson–Bailey class host/parasitoid population model parameterized with data from the experiments above to simulate interactions between *A*. *certus* and the two aphid species. The basic model and some variations have been described previously (Heimpel et al., 2003; Kaser & Heimpel 2015; Abram et al., 2016; Kaser et al., 2018); the modeling here is meant to be heuristic in nature rather than strictly predictive given the relative simplicity of these classes of population models (Hassell, 2000).

The model followed populations of one parasitoid species (*P*), suitable host species (*H*1), and an unsuitable host species (*H*2):

$$\begin{array}{c} H1_{t+1}=H1_{t}*g\left(H1_{t}\right)*\left[1‐\left(s_{1}+\mu_{1}\right)*(1‐f\right]∙1{,}_{t}\left[)\right]\\ H2_{t+1}=H2_{t}*g\left(H2_{t}\right)*\left[(1‐\left(s_{2}+\mu_{2}\right)*(1‐f\right]∙2{,}_{t}\left[)\right]\\ P_{t+1}=X*\left(s1*H1_{t}*\left[1‐f\left(∙1{,}_{t}\right)\left[+s2*H2_{t}*\right]1‐f\left(∙2{,}_{t}\right)\right]\right) \end{array}$$

where

$$g\left(H_{i,t}\right)=\exp\left[r_{i}\left(1‐\frac{H_{i,t}}{K_{i}}\right)\right],$$

$$f\left(\varepsilon_{i,t}\right)=\left(1+\frac{\varepsilon_{i,t}}{k}\right){,}^{‐k}$$

and

$$\varepsilon_{i,t}=\frac{a_{i}\beta P_{t}}{\beta +a_{1}H_{1,t}+a_{2}H_{2,t}}.$$

At time *t*, the number of suitable hosts (*H*1, *A*.* glycines*), the number of unsuitable hosts (*A*.* nerii*; *H*2) and the parasitoid by *P (Aphelinus certus)*. The function *g*(*Hi_t_*) of host species *i* is the fraction of hosts surviving given competition‐driven density‐dependent survival among host individuals and depends on the intrinsic rate of growth of hosts denoted *r*_i_, the carrying capacity of hosts *K*_i_ and the number of hosts at time *t* denoted *H*_i, t_ (Equation set (2)). The function *f*(*ε*_i,t_) is the proportion of host population *i* that does not encounter a parasitoid and depends on *k* as the risk of aggregated parasitism, *a_i_* as the parasitoid search rate, *β* as the maximum parasitoid fecundity (related to the saturation of the functional response incorporating information about egg and time limitation), and *P_t_* as the number of parasitoids at time *t* (Heimpel et al., 2003, Equation set (2)). The egg load parameter *β* sets the saturation of the functional response and can thus be interpreted as indicating egg limitation and/or time limitation (Getz & Mills, 1996). *s*_i_ is the susceptibility of hosts to parasitism (i.e., the proportion of parasitized hosts enabling offspring production; more details are available in Godfray & Hassell, 1991 and Heimpel et al., 2003). The parameter *µ_i_* denotes the proportion of parasitized hosts killed by the parasitoid without producing parasitoid offspring as in Abram et al. (2016) and Kaser et al. (2018). Both *s*_i_ and *µ_i_* vary between 0 and 1. Suitable hosts have a *s*_i_ of 1 and a *µ_i_* close to 0, whereas completely unsuitable hosts have a *s*_i_ of 0 and a *µ_i_* between 0 and 1 depending on the nonreproductive mortality induced by the parasitoid. We also introduce a new parameter to denote sex ratio: We define *X* as the percentage of viable adult parasitoids that are females at the beginning of a given generation.

The parameters *a*, β, *X*, *s*_i,_ and *µ_i_* were estimated for the parasitoid *Aphelinus certus* and the hosts *Aphis glycines* and *A*. *nerii* using the experiments of this study. *K*_i_ and *k*_i_ were fixed at 1,000 and 0.75, respectively (see discussion about sensitivity analysis of risk of aggregated parasitism in Heimpel et al. (2003)). All simulations were run for 100 generations to make sure a stable equilibrium or limit cycles is reached using R software version 3.3.3 (R Core Team, 2017).

### 3.2 Statistical analyses

The correlation and relationship between the egg load and the tibia length of the dissected parasitoids in experiment 1.1 were determined using a Pearson's correlation test and a linear regression model, respectively. A series of analyses of variance (ANOVA) were used to test for effects of the choice or no‐choice setting on the number of (i) eggs deposited in the aphids, (ii) mummies produced on the two aphid species, and (iii) eggs remaining within parasitoid females in experiment 1.1. The use of ANOVAs for these tests was appropriate since the dependent variables followed a normal distribution using a Shapiro–Wilk test and since visual interpretation of quantile–quantile plots also suggested adherence to a normal distribution. The effect of the aphid species on all of the previously mentioned dependent variables in both the no‐choice and choice situations as well as the impact of the aphid density on all of the previously cited variables in the choice situation were also analyzed using ANOVA. The effects of the choice/no‐choice setting on the number of eggs remaining within parasitoid females in experiment 1.2, and the effect of aphid density on remaining eggs in the choice setting were evaluated using separate generalized linear models (GLM) with a Poisson error distribution and testing the significance with a chi‐square test. The effect of the choice/no‐choice setting on the parasitoid emergence rate (number of emerged parasitoid/ number of mummies) and on the female sex ratio (number of females/ total number of emerged parasitoids) were compared for parasitoids that produced ≥ 10 mummies (the number deemed necessary to reasonably estimate the emergence rate and the sex ratio) were assessed using a generalized linear model with a binomial distribution as were the effects of aphid density on these independent variables in the choice situation.

The effect of parasitism on aphid longevity in Experiment 2 was compared by fitting a Cox proportional hazards model and testing the significance with a Score (logrank) test (using the package “survival” in R version 3.3.3). The effect of parasitism on the cumulative number of nymphs deposited and the pre‐oviposition period (in days) of the milkweed aphid were compared using a generalized linear model with a Poisson distribution of errors and testing the significance of the variables with a chi‐square test. All statistical analyses were done using R. 3.3.3.

## 4 RESULTS

### 4.1 Experiment 1: Parasitoid‐mediated indirect interactions between milkweed aphid and soybean aphid

#### 4.1.1 Experiment 11: Parasitoid oviposition

**Figure 1:**
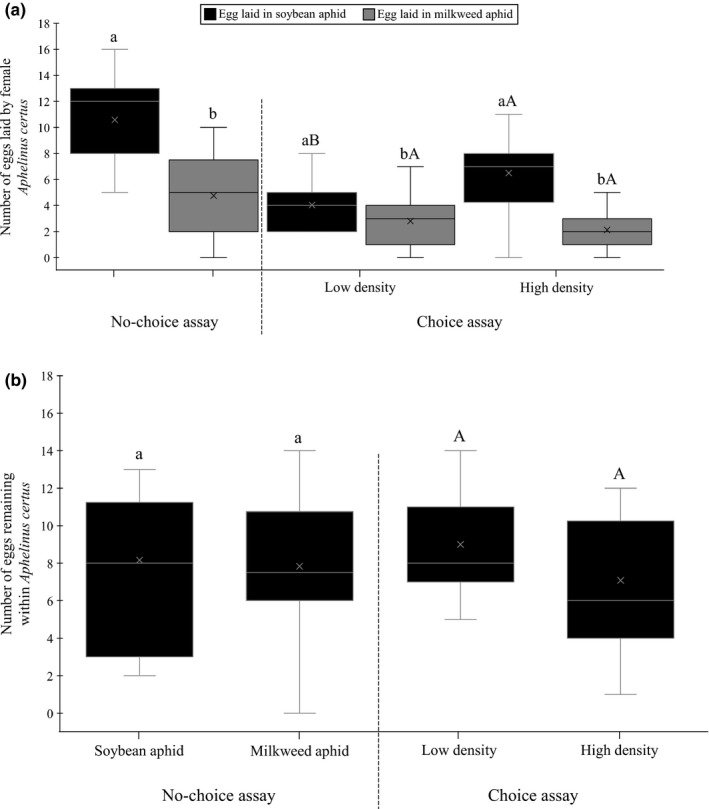
Number of (a) eggs laid in soybean aphid or milkweed aphid by the parasitoid *A*.* certus* and (b) eggs remaining within parasitoid females, after 24 hr of parasitism in the four different treatments. In the no‐choice assay, boxplot followed by the same lower case letter did not differ statistically. In the choice assays, boxplot followed by the same lower case letter did not differ significantly with respect to host density, and boxplot followed by the same upper‐case letter did not differ significantly with respect to host species

The number of eggs laid in soybean aphids or milkweed aphids varied significantly depending on whether a choice of host species was offered or not (*F*_1; 75_:66.93, *p* < .001 for soybean aphid; *F*_1, 73_:16.17, *p* < .001 for milkweed aphid; Figure 1a). *Aphelinus certus* females laid 2.2 and 2.1 times more eggs in soybean aphids than in milkweed aphids in both no‐choice (*F*_1, 50_:45.33, *p* < .001; Figure 1a) and choice settings, respectively, and in the latter in the presence of both low and high aphid densities (*F*_1; 46_:50.64, *p* = .024; *F*_1, 50_:5.44, *p* < .001, respectively; Figure 1a). In the choice assay, the number of eggs laid in soybean aphid was 1.6 times higher at high than at low host density (*F*_1, 48_:16.14, *p* < .001) whereas the number of eggs laid in milkweed aphid did not vary significantly depending on aphid density (*F*_1, 48_:1.70, *p* = .199). The number of eggs remaining within female parasitoids after the experiment was not significantly different in no‐choice versus choice situations (soybean aphid: *F*_1; 34_:0.01, *p* = .945; milkweed aphid: *F*_1; 33_:0.07, *p* = .795) and also did not vary significantly depending on the aphid species encountered (*F*_1, 21_:0.05, *p* = .820) or the aphid density (*F*_1, 22_:1.24, *p* = .277) (Figure 1b).

**Figure 2:**
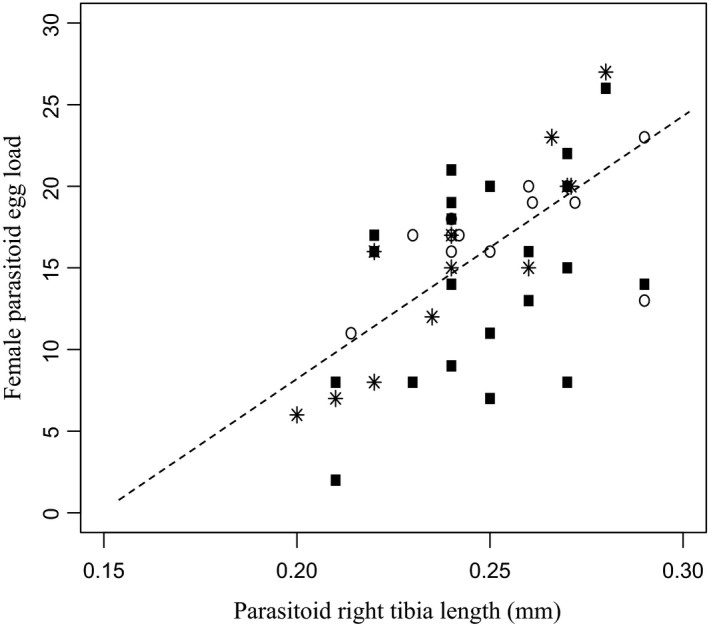
Female parasitoid egg load as a function of right tibia length across treatments (*t*_45_ = 5.36, *R* ^2^ = 0.39, *p* < .001) for the no‐choice (black square) and choice experimental settings and the aphid density in the choice setting (empty circle: low‐density, asterisks: high‐density). The dashed line represents the linear equation between the egg load (Y) and the tibia length (x) of the female parasitoids: *Y = 164*.*79x − 24*.*68*

*Aphelinus certus* hind tibia length was not significantly different between females assigned to the choice and no‐choice treatments (soybean aphid: *F*_1, 34_:1.05, *p* = .312; milkweed aphid: *F*_1, 33_:0.31, *p* = .582) or between females assigned to the high‐ and low‐density choice situations (*F*_1, 22_:0.92, *p* = .349) (Figure 2). In addition, the egg load and the tibia length of females used in the experiment were strongly correlated (*t*_45_ = 5.36, *R* ^2^ = 0.39, *p* < .001; Figure 2).

#### 4.1.2 Experiment 1.2: Parasitoid developmental success and offspring fitness

**Figure 3:**
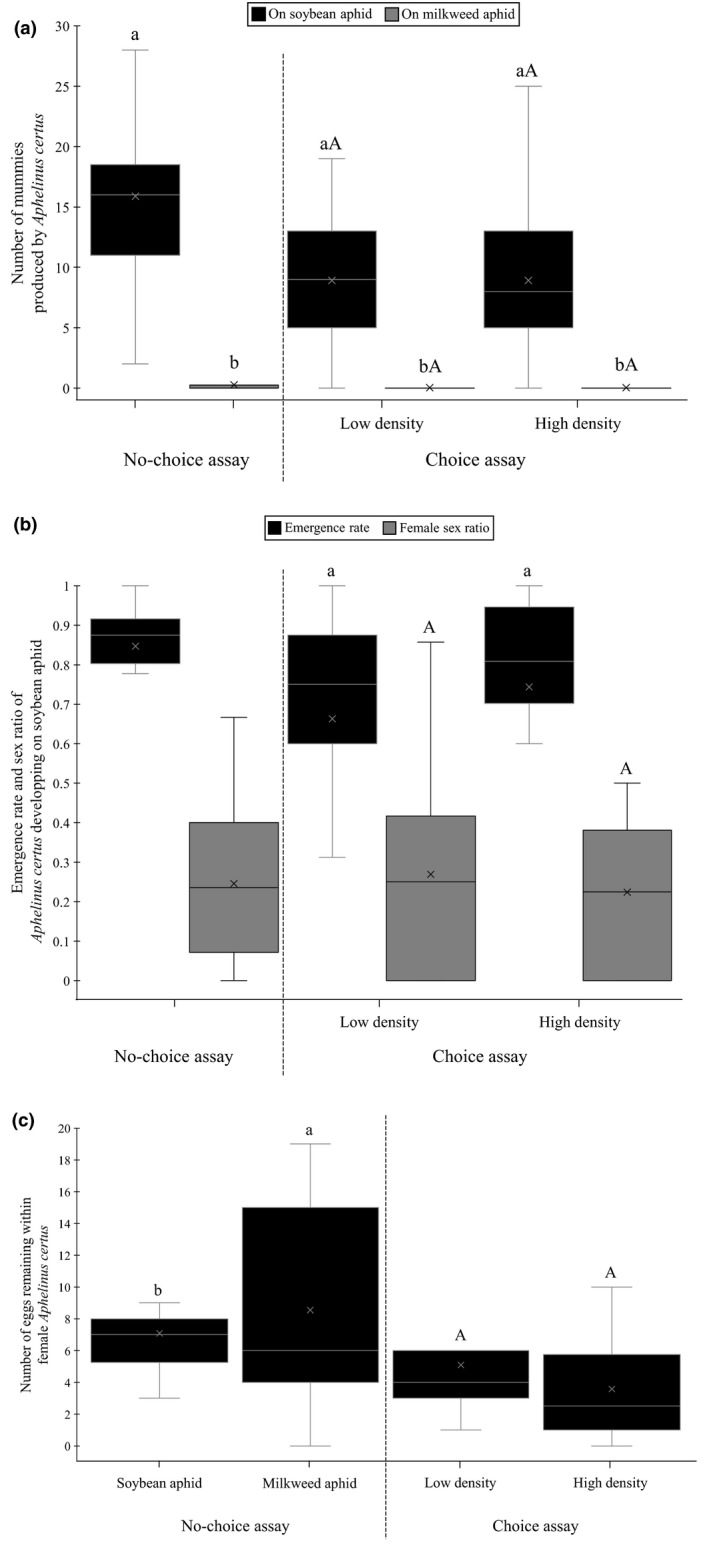
Number of (a) soybean aphids and milkweed aphids mummified by *A*.* certus*, (b) adult parasitoid emergence rate and female sex ratio of offspring when developing on soybean aphid, and (c) eggs remaining within females after 24 hr of parasitism in the four different treatments. In the no‐choice assay, boxplot followed by the same lower case letter did not differ. In the choice assays, boxplot followed by the same lower case letter did not differ significantly with respect to host density, and boxplot followed by the same upper‐case letter did not differ significantly with respect to host species

*Aphelinus certus* produced 1.7 and 7 times more mummies under the no‐choice than the choice setting when encountering soybean aphid or milkweed aphid (*F*_1; 84_:25.43, *p* < .001; *F*_1; 69_:7.09, *p* = .009, respectively), leading to significantly more mummies on soybean aphid than on milkweed aphid in the choice (*F*_1, 49_:96.88, *p* < .001; Figure 3a) and in the choice test under both high and low aphid densities (*F*_1, 50_:56.33, *p* < .001; *F*_1; 52_:71.4, *p* < .001, respectively; Figure 3a). Within the choice test, the number of mummies produced on soybean aphid or milkweed aphid was not significantly affected by aphid density (*F*_1; 51_:0.01, *p* = .999; *F*_1; 51_:0.01, *p* = .979, respectively; Figure 3a).

The number of parasitoid mummies produced on milkweed aphid was too low to reasonably estimate the emergence rate or the sex ratio and so we report only results associated with soybean aphid. The parasitoid emergence rate and the female sex ratio produced on soybean aphid did not differ significantly between the choice and no‐choice setting (χ^2^_1_: 0.39, *p* = .530; χ^2^_1_: 6.148.4, *p* = .076057, respectively) and did not vary with aphid density in the choice setting (χ^2^_1_: 1.33, *p* = .248; χ^2^_1_: 3.65, *p* = .156) (Figure 3b). After the experiment, there were significantly more remaining eggs (1.7 times more) within females in no‐choice than in choice settings (soybean aphid: χ^2^_1_: 11.1, *p* < .001; milkweed aphid: χ^2^_1_: 22.1, *p* < .001) (Figure 3c). The number of remaining eggs did not vary significantly within females exposed to milkweed aphid or soybean aphid in the no‐choice assay (χ^2^_1_: 1.6, *p = *.210; Figure 3c) and within females exposed to different aphid densities in the choice assay (χ^2^_1_: 6.1, *p* = .154).

### 4.2 Experiment 2: Impact of parasitism on milkweed aphid

**Figure 4:**
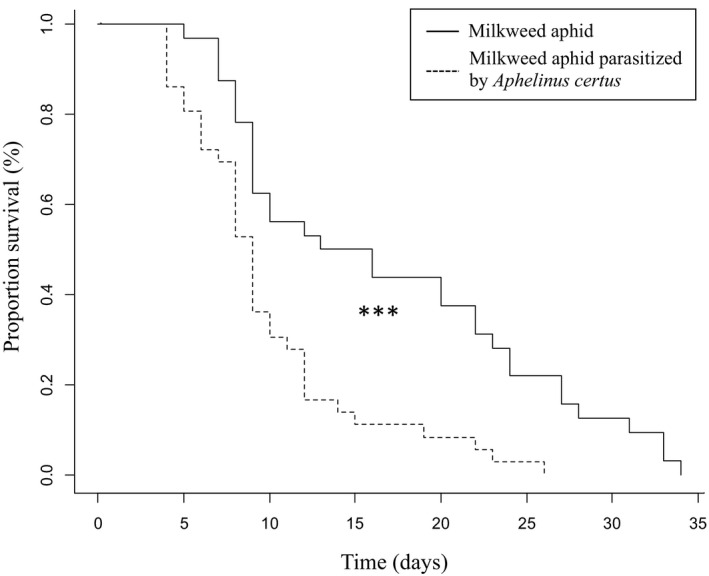
Survival curves of milkweed aphids when parasitized by *A*.* certus* compared to control. ***Indicates that the two curves are statistically different (*p* < .001)

The longevity of milkweed aphid was 1.4 times lower when parasitized by *A*. *certus* than when not parasitized (score (logrank) test = 12.32 on 1 *df*, *p* < .001, Figure 4). Similarly, the milkweed aphid fecundity was 2.5 times lower when parasitized by *A*.* certus* (12.2 ± 2.9 offspring produced) than when not parasitized (30.7 ± 4.5 offspring produced) (X^2^_1_: 282.82, *p* < .001). By contrast, the period of time before the first nymphs were laid by milkweed aphid stung by the parasitoid was not significantly different from those not stung and took on average 7 days (X^2^_1_: 0.046, *p* > .05).

### 4.3 Impact of interactions on host–parasitoid population dynamics

The parameter values used in the model were 1 for *a*_1_, 0.45 for *a*_2_ (estimated from experiment 1.1), 16 for *β* (estimated from experiment 1.1 and Appendix A), 0.29 for *X* (estimate from experiment 1.2), 1 and 0 for *s*_1_ and *s*_2_, respectively, and 0 for *µ*_1_ considering soybean aphid is a highly suitable host for *A*.* certus*. In this study, the parasitoids did not kill the unsuitable hosts but had a negative impact on their fitness; milkweed aphid fecundity was reduced by 40% when they were parasitized by the parasitoid (Experiment 2). Hence, *µ*_2_ = 0.4.

**Figure 5:**
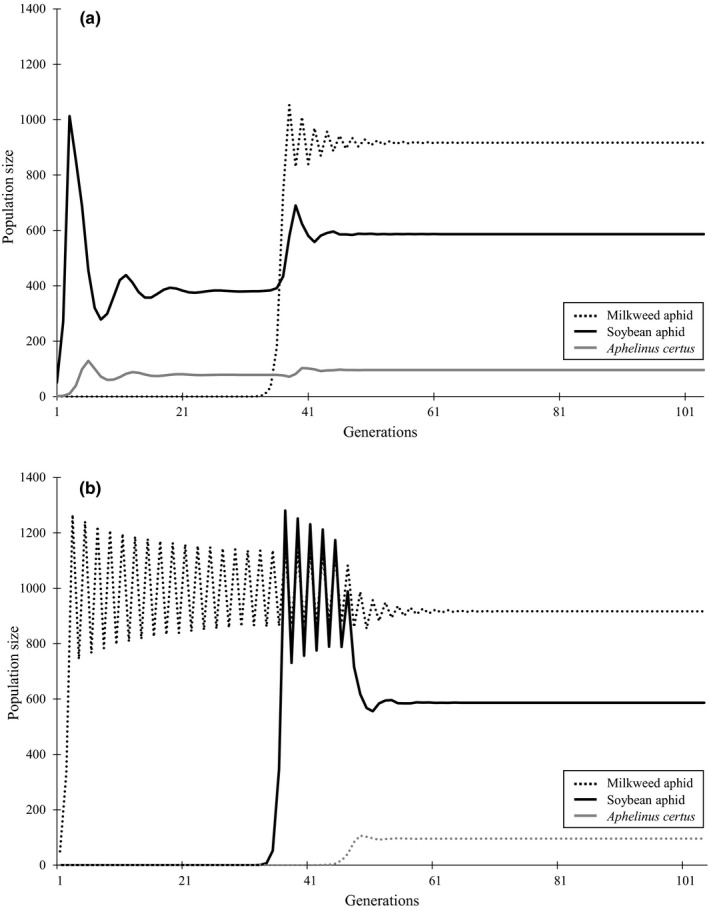
A simulation using the model in which (a) the marginal host invades an ongoing interaction between the suitable host and the parasitoid and (b) milkweed aphid is present first, and soybean aphid and *A*. *certus* invade. Model parameters are *a_1_* = 1, *a_2_* = 0.45, *r*_1_ = *r*_2_= 2, *β* = 16, *s_1_ = *1, *s_2_* = 0, *µ*_1_ = 0, *µ*_2_ *= *0.4, *k* = 0.75, *K_i_* = 1,000, *X* = 0.29, *H_1_ = H_2_*= 50 and *p* = 1

When milkweed aphid invades a situation where soybean aphid and *A*.* certus* are at their equilibrium levels (after 30 generations) at the default parameter levels, it quickly reaches a population exceeding its carrying capacity and settles to an equilibrium population size after about 15 generations of decreasing limit cycles (Figure 5a). Meanwhile, population sizes of both soybean aphid and *A*. *certus* increase by factors of 1.54 and 1.23, respectively. Milkweed aphid populations alone cannot support a population of parasitoids, and alone, there populations cycle indefinitely around the carrying capacity of 1,000 (Figure 5b). Therefore, the presence of the soybean aphid does not have a direct effect on the milkweed aphid population but an indirect effect by supporting populations of *A*.* certus*. When the soybean aphids and *A*.* certus* are introduced into such a population of milkweed aphids, their population drops by approximately 10% to a single equilibrium of 916, and populations of soybean aphids and parasitoids reach the same equilibrium levels as in the previous simulation. Thus, the presence of the unsuitable host (milkweed aphid) increases populations of the suitable host (soybean aphid) but the presence of soybean aphid decreases populations of milkweed aphid, and both of these interactions are mediated by the shared parasitoid *A*. *certus*. This pattern of indirect interactions (+/−) can be termed “apparent predation” after Holt (1977).

## 5 DISCUSSION

We found that the parasitoid *Aphelinus certus* parasitized fewer individuals of the soybean aphid (a suitable host) when in the presence of milkweed aphid, an unsuitable host. Aphid dissections showed that parasitoid eggs were laid into milkweed aphids despite this being a very poor host for parasitoid development. Thus, milkweed aphid was acting as an “egg sink” for the parasitoid and this left fewer available eggs for deposition into soybean aphid. A population model incorporating parameters gathered in these laboratory studies was consistent with an egg‐ and/or time‐sink‐mediated indirect (+/−) interaction (apparent predation; Holt, 1977) between these two aphid species. This class of interactions had been hypothesized for interactions in which individuals a single parasitoid species attack host species of varying suitability (Heimpel et al., 2003) and various of the empirical requirements for such interactions have been observed in parasitoids of adult ladybeetles and insect eggs (Hoogendoorn & Heimpel, 2002; Abram et al., 2014, 2016; Kaser et al., 2018). As far as we are aware, this is the first report demonstrating apparent predation mediated by an aphid parasitoid.

In order for egg sinks to impact population dynamics, parasitoids must experience egg limitation and/or time limitation associated with the handling of the unsuitable host (Abram et al., 2016; Heimpel et al., 2003). Our experiment provided little evidence of absolute egg limitation (i.e., egg loads of zero) as parasitoid dissections revealed averages of between four and eight eggs at the end of trials depending on experimental treatments. *Aphelinus* species are known to mature eggs rapidly (Hopper et al., 2013; Le Ralec 1995; Wu & Heimpel, 2007) so absolute egg limitation would likely be transient in any case. However, parasitoids also tend to decrease their oviposition rate as egg loads decline as a means of avoiding absolute egg limitation (Heimpel & Rosenheim, 1998; Mangel & Heimpel, 1998; Minkenberg et al., 1992). Thus, situations close to egg limitation may have contributed to lower oviposition rates into soybean aphid in the presence of milkweed aphid even without actual absolute egg limitation. It is also not unreasonable to posit that time limitation could have contributed to low oviposition rates in soybean aphid in our trials. *Aphelinus* spp. tend to have relatively long handling times that includes using some hosts for host feeding (e.g., Bai & Mackauer, 1990; Miksanek & Heimpel, 2020b) and the time taken handling milkweed aphid could have restricted time available for oviposition into soybean aphid sufficiently to reduce oviposition rates overall.

The result that we found is a result of parasitoids ovipositing into individuals of an unsuitable host species—a behavior that may be considered a “mistake.” However, the females of many parasitoid species attack unsuitable or poorly suitable hosts (Heimpel et al., 2003) and such behavior may be adaptive for parasitoids that carry many eggs and yet are limited by the time available to oviposit (Heimpel & Rosenheim, 1998; Minkenberg et al., 1992). Such a scenario does not seem to be a likely explanation for acceptance of the milkweed aphid by *A*.* certus* in our study, however. This parasitoid species has a relatively low egg load (see also Hopper et al., 2013; Monticelli et al., 2019a) and our dissections from the choice studies clearly show that eggs that could have been laid into the suitable soybean aphid were instead laid into the unsuitable milkweed aphid. We believe rather that *A*.* certus* attacks unsuitable hosts as reflection of the fact that it is a generalist aphid parasitoid (Hopper et al., 2017a). In this case the “mistake” of attacking *A*.* nerii* represents the outcome of the classical trade‐off faced by generalists: the ability to develop in many host species leads to weak selection for discrimination ability between suitable and unsuitable hosts and thus some unsuitable hosts are inevitably attacked (Asplen et al., 2012). Trade‐offs between host‐range and host‐use efficiency have been identified in aphid parasitoids in the braconid subfamily Aphidiinae (Straub et al., 2011), and our results suggest that they may be operating in *Aphelinus* species as well. An evolutionary trap, that is, a sudden environmental change that de‐couples an acceptance cue from the resulting fitness payoff due to a lack of shared evolutionary history (Schlaepfer et al., 2002, 2005), may also be invoked to explain acceptance of unsuitable hosts by parasitoids as *A*.* certus* was introduced from Asia to North America in 2005 (Heimpel et al., 2010; Hopper et al., 2019).

Although milkweed aphid is unsuitable for the development of *A*.* certus*, it still suffers fitness costs of being parasitized. Dissections showed that the immature parasitoids developing in milkweed aphid died at the larval stage, presumably owing to their inability to detoxify toxic compounds (cardenolides) sequestered by the aphids (Desneux et al., 2009a; Jeschke et al., 2016; Monticelli et al., 2019a; Mooney et al., 2008). We hypothesize that the activities of the immature parasitoids prior to their deaths have consequences on host fitness due to energy costs (Desneux et al., 2009b; Godfray, 1994; Graham et al., 2011; Schmitz et al., 2012; Strand, 2014) resulting in lower longevity and fecundity. Abram et al. (2016) added variable fitness of unsuitable hosts in their updating of the model introduced by Heimpel et al. (2003), and we present here the first application of this updated model to an aphid/parasitoid system. We showed that the fecundity of attacked milkweed aphids was reduced by 40% when both soybean aphids and *A*. *certus* were present (Experiment 2), leading to a 10% reduction in milkweed aphid population size (Figure 5) since parasitized aphids were still able to produce 10 offspring per individual.

Milkweed aphid is a holarctically distributed aphid species that is unsuitable for a number of parasitoids that nevertheless attack it (Desneux et al., 2009a; Monticelli et al., 2019a). Thus, the role played by milkweed aphid in our study may be generalized to other systems in which hosts of milkweed aphid (milkweed plants in North America and oleander in Europe) grow adjacent to other plants with suitable aphid hosts. In North America, this aphid has been found on milkweed plants growing inside and outside soybean field (Martin & Burnside, 1980) and *A*.* certus* is a biological control agent of the soybean aphid in both the United States and Canada (Frewin et al., 2010; Hallett et al., 2014; Kaser & Heimpel, 2018; LeBlanc & Brodeur, 2018; Miksanek & Heimpel, 2019). The presence of milkweed aphid within and adjacent to soybean fields in North America may therefore interfere with biological control of soybean aphid by *Aphelinus certus*.

While the dynamics considered in this study involved situations in which the aphids were growing on different host species, a similar egg sink effect could occur on a single host plant supporting both suitable and unsuitable species (as can occur with multiple aphid species on milkweed for example; Smith et al., 2008). Beyond this, egg sinks could occur within a single host species if there is intraspecific variability in host suitability, as can happen in aphid systems due to differences in the genetic basis of resistance to parasitoids (Martinez et al., 2014) or to differences in the presence of defensive endosymbionts (Asplen et al., 2014; Desneux et al., 2018; Monticelli et al., 2019b; Oliver et al., 2014). Our results demonstrated that the presence of a lower‐quality host can reduce the effectiveness of a parasitoid as a biological control agent but also reduce the fitness of alternative hosts not targeted by biocontrol programs. Additional research is needed to determine the impact of the presence of these unsuitable hosts on the control of pest populations in the field.

## CONFLICT OF INTEREST

The authors declare no conflict of interest.

## AUTHOR CONTRIBUTION

**Lucie Monticelli:** Conceptualization (equal); Data curation (lead); Formal analysis (lead); Writing‐original draft (lead). **Nicolas Desneux:** Conceptualization (equal); Supervision (equal); Writing‐review & editing (supporting). **George Heimpel:** Conceptualization (equal); Supervision (equal); Writing‐review & editing (lead).

## Supporting information

Supplementary Material

## Data Availability

Monticelli, Lucie (2021), Parasitoid‐mediated indirect interactions between unsuitable and suitable hosts can generate apparent predation in microcosm and modeling studies, v2, Dryad, Dataset, https://doi.org/10.5061/dryad.02v6wwq1p.
