# Supplementary material for: Parasitoid‐mediated indirect interactions between unsuitable and suitable hosts generate apparent predation in microcosm and modeling studies
Source: Ecol Evol. 2021 Feb 23;11(6):2449–60. doi: 10.1002/ece3.6896 (PMC7981237; doi:10.1002/ece3.6896)

Appendix A. Impact of host feeding on *A. certus* egg number.

Egg production in *A. certus* may vary with the food taken by adult females (Le ralec 1995, Olson et al. 2005). Hence, we compared the number of eggs produced by the parasitoid depending on the food provided. Naive emerged female parasitoids (<12 hour-old) were placed in 0.6mL microcentrifuge tubes with (1) water (W, n=21), (2) water and honey (H, n=26), (3) water and one soybean aphid individual (AG, n=20) or (4) water and one milkweed aphid individual (AN, n=22) for 24 hours before dissecting the females at 40x magnification to count the number of mature eggs. Aphids were considered consumed when they were flattened, orange/pink and when after dissection (x40), no egg was found in it. The number of eggs was compared among the treatments (W, H, AG and AN) using a generalized linear model with Poisson distribution. Bars with the same letter are not significantly different (multi-comparison test of the package 'multcomp'). Statistical results: X^2_3: 21.57, $P < 0.001$.

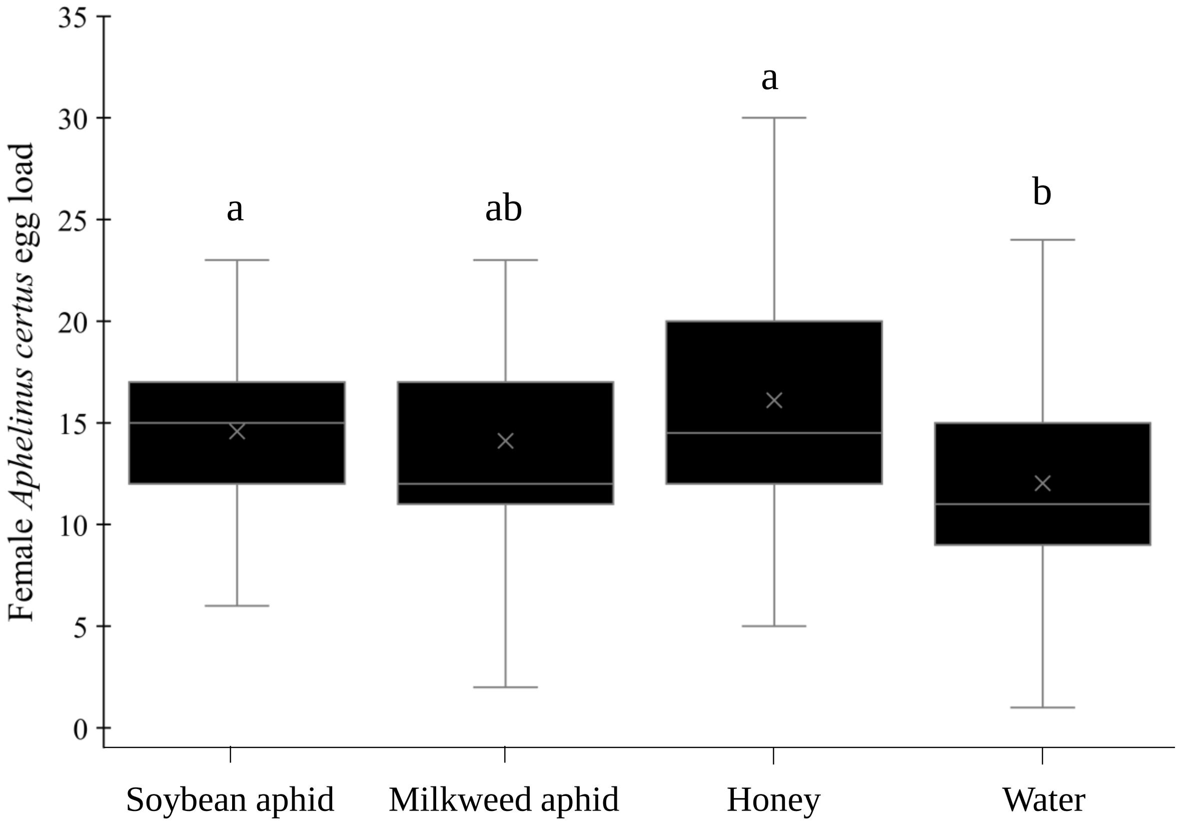

Supplement: Supplementary file 1 — Supplementary Material [file ECE3-11-2449-s001.pdf]
